# Diagnostic Accuracy of Severe Acute Respiratory Infection Definitions in Hospitalized Children: A Systematic Review and Meta-Analysis

**DOI:** 10.1001/jamanetworkopen.2025.50298

**Published:** 2025-12-18

**Authors:** Leo Hersi, Tuana Kant, Caitlyn L. Kaziev, Daniel S. Farrar, Noah Bryan, Jessie Cunningham, Haifa Mtaweh, Sanjay Mahant, Shaun K. Morris, Peter J. Gill

**Affiliations:** 1 School of Medicine, Queen’s University, Kingston, Ontario, Canada; 2 Child Health Evaluative Sciences, SickKids Research Institute, Toronto, Ontario, Canada; 3 Temerty Faculty of Medicine, University of Toronto, Toronto, Ontario, Canada; 4 Centre for Global Child Health, The Hospital for Sick Children, Toronto, Ontario, Canada; 5 Health Sciences Library, The Hospital for Sick Children, Toronto, Ontario, Canada; 6 Division of Paediatric Medicine, The Hospital for Sick Children, Toronto, Ontario, Canada; 7 Department of Paediatrics, University of Toronto, Toronto, Ontario, Canada; 8 Institute of Health Policy, Management, and Evaluation, University of Toronto, Toronto, Ontario, Canada; 9 Division of Paediatric Infectious Diseases, The Hospital for Sick Children, Toronto, Ontario, Canada; 10 Dalla Lana School of Public Health, University of Toronto, Toronto, Ontario, Canada

## Abstract

**Importance:**

Following the 2009 H1N1 influenza pandemic, the World Health Organization (WHO) established a new case definition for severe acute respiratory infection (SARI) for viral surveillance. Several studies have suggested that SARI case definitions are inaccurate at detecting pediatric disease burden. Understanding the performance of SARI case definitions in children is important for pandemic preparedness.

**Objectives:**

To evaluate the diagnostic accuracy of SARI case definitions in detecting microbiologically confirmed viral respiratory tract infections among hospitalized children.

**Data Sources:**

The MEDLINE(R), Embase Classic + Embase, Ovid EBM Reviews Cochrane Central Register of Controlled Trials, Elsevier SCOPUS, and the WHO Global Index Medicus databases were searched from inception to March 31, 2025.

**Study Selection:**

Study screening was conducted in duplicate by 2 independent reviewers. Any studies that assessed any SARI definition in hospitalized children were included. There were no restrictions by design, time period, or geographical location.

**Data Extraction and Synthesis:**

Data extraction using the Preferred Reporting Items for Systematic Reviews and Meta-Analyses reporting guideline was conducted by 1 author using a predefined template and independently validated by a second author. Diagnostic accuracy was extracted as 2 × 2 tables from each study and pooled using a bivariate random-effects model. Quality assessments were conducted using the Quality Assessment of Diagnostic Accuracy Studies (QUADAS-2) tool.

**Main Outcomes and Measures:**

The primary outcomes were sensitivity and specificity. For each case definition–virus combination with at least 4 included studies, pooled estimates of sensitivity and specificity were calculated.

**Results:**

Of 1144 studies identified, 13 were included. Included studies represent surveillance data from 65 inpatient sites across 8 countries, using data from 2007 to 2023. The most common definition was the 2014 WHO SARI (9 studies). Viral pathogens included influenza (10 studies) and respiratory syncytial virus (RSV; 6 studies). Meta-analysis of the WHO 2014 SARI definition yielded a sensitivity of 75.7% (95% CI, 65.0%-83.9%; *I*^2^ = 89.2%) and specificity of 30.6% (95% CI, 19.8%-44.0%; *I*^2^ = 99.0%) for influenza (7 studies) and sensitivity of 70.6% (95% CI, 56.9%-81.9%; *I*^2^ = 98.8%) and specificity of 38.7% (95% CI, 25.7%-53.5%; *I*^2^ = 99.5%) for RSV (5 studies). In younger subgroups, sensitivity appeared to decrease while specificity appeared to increase for both influenza and RSV.

**Conclusions and Relevance:**

In this systematic review and meta-analysis of 13 studies, the WHO 2014 SARI definition demonstrated reduced sensitivity and increased specificity in younger pediatric cohorts, suggesting that surveillance systems that rely on SARI case definitions may potentially underestimate disease burden in children.

## Key Points

**Question:**

How accurate are existing case definitions for severe acute respiratory infection (SARI) in detecting viral respiratory tract infections in hospitalized children?

**Findings:**

This systematic review and meta-analysis of 13 studies found the World Health Organization 2014 SARI case definition to be the most widely used. Meta-analysis yielded a pooled sensitivity and specificity of 75.7% and 30.6% for influenza and 70.6% and 38.7% for respiratory syncytial virus, respectively; sensitivity decreased greatly in younger age groups.

**Meaning:**

These findings suggest that existing viral surveillance resources that rely on SARI case definitions may underestimate disease burden in young pediatric cohorts.

## Teaser

This systematic review and meta-analysis evaluates the diagnostic accuracy of severe acute respiratory infection (SARI) case definitions for detecting microbiologically confirmed viral respiratory tract infections among hospitalized children.

## Introduction

Severe acute respiratory infection (SARI) surveillance systems were established to monitor patterns in severe respiratory disease and support the early detection of novel respiratory strains.^1,2^ By contributing to the recognition of unusual patterns, robust sentinel surveillance systems remain essential for public health system planning and pandemic preparedness.^1,3^ In 1999, the World Health Organization (WHO) first recommended a clinical case definition for influenza-like illness surveillance in individuals older than 5 years.^3^ The definition classified suspected cases as those with a sudden onset of fever of greater than 38 °C and cough or sore throat, in the absence of other diagnoses.^1^ For children younger than 5 years, WHO recommended using the Integrated Management for Childhood Illness (IMCI) definitions of pneumonia and severe pneumonia, originally intended for clinical care by community health workers in low- and middle-income countries, for surveillance purposes as well.^4,5^

The 2003 severe acute respiratory syndrome outbreak and 2009 H1N1 influenza pandemic revealed inadequacies in international, national, and subnational surveillance for novel respiratory pathogens with pandemic potential, prompting a global reassessment of the necessary resources for widespread viral surveillance.^4,6^ Discrepancies in reporting practices between public health and clinical systems, both within and between countries, resulted in data incompatibility at the frontline of pandemic response, which created difficulties in rapidly understanding the epidemiology of these infections. Further, the IMCI definition—the only widely used surveillance definition for young children before 2011—was not originally designed for surveillance and was often deemed complicated for clinical settings.^5^ In 2011, the WHO introduced a new standardized surveillance case definition for SARI,^4^ which consisted of a history of fever or measured fever of greater than 38 °C, cough, symptom onset within the last 7 days, and requiring hospitalization.^4^ The new definition, notably designed to include both children and adults, aimed to capture the severe influenza-related pneumonias and influenza-related exacerbations of chronic disease, marking a substantial shift from the pre-2011 consultation case definitions.^7^ In 2014, the definition was revised to extend the symptom onset window from 7 to 10 days.^3,8^

The WHO 2014 SARI definition was designed to strike the balance between sensitivity and specificity to estimate disease impact.^4,7^ Several studies have evaluated the performance of the WHO 2014 SARI definition in detecting viral respiratory infections in adult inpatients. Against influenza, diagnostic accuracy studies have reported sensitivities ranging between 78.8% to 91.9% and specificities ranging between 18.0% to 32.5% in adult cohorts.^9,10,11^ Against RSV, a systematic review comparing several case definitions found that the WHO 2014 definition had a sensitivity ranging between 78.8% to 89.0% and specificities ranging between 6.6% to 27.7% in adults aged 18 to 64 years.^9,12,13^ However, several studies have reported that the SARI criteria have suboptimal performance in detecting severe respiratory viral infections in young children.^8,14,15,16^ Given the importance of respiratory tract infections in children, due to their role as viral vectors and the impact on morbidity and mortality, evaluating the diagnostic accuracy of the SARI criteria in pediatric cohorts is of particular importance.^14,17^ To date, there have been no systematic reviews assessing the diagnostic accuracy of the SARI definition specifically among pediatric populations, nor against multiple viruses. Therefore, the objective of our study was to conduct a systematic review to evaluate the diagnostic accuracy of existing SARI surveillance case definitions in detecting microbiologically confirmed viral acute respiratory tract infections among hospitalized children.

## Methods

### Study Registration

The systematic review and meta-analysis was registered at the International Prospective Register of Systematic Reviews (CRD42024557706). The study was reported in accordance with the Preferred Reporting Items for Systematic Reviews and Meta-Analyses (PRISMA) reporting guideline.^18^

### Search Strategy and Eligibility Criteria

A reference librarian (J.C.) designed and conducted an electronic search strategy on Ovid MEDLINE(R) and Epub Ahead of Print, In-Process & Other Non-Indexed Citations and Daily, Ovid Embase Classic + Embase, Ovid EBM Reviews Cochrane Central Register of Controlled Trials, Elsevier SCOPUS, and the WHO Global Index Medicus on June 11, 2024. The search was updated on March 31, 2025. The search strategy is included in the eMethods in Supplement 1. We also searched national organization websites for sentinel surveillance reports matching inclusion criteria. Reference lists of eligible studies were further searched.

We included studies which (1) evaluated the sensitivity and specificity of any SARI surveillance case definition, (2) were conducted among cohorts aged younger than 18 years, and (3) verified viral respiratory infections with any assay, including real-time reverse transcription polymerase chain reaction (rRT-PCR) or rapid antigen testing. Outpatient surveillance criteria, such as influenza-like illness, were excluded. Studies were included if they reported diagnostic accuracy as sensitivity and specificity directly or provided sufficient raw data to allow for their calculation. Studies were included if they reported age-stratified data resulting in a distinct, extractable pediatric cohort. No restrictions were placed on study design, geographic location, or study period. Studies in languages other than English were translated where possible. Studies published only as posters or abstracts were excluded due to insufficient methods and data for appropriate comparison.

### Selection and Data Collection

Title and abstract screening, followed by full text screening, were conducted in duplicate using Covidence by at least 2 independent reviewers (L.H., N.B., and T.K.) with conflicts resolved by a third author (P.J.G.). Data extraction was completed by one author using a predefined Microsoft Excel template and validated by a second author. Extracted variables included the author, population, case definitions used, SARI case definition, viruses of interest, and diagnostic accuracy. Data reported in supplementary datasets were extracted and analyzed using R version 4.4.1 (R Project for Statistical Computing).^19^ When pertinent data were missing, we contacted the corresponding authorship teams, who provided either complete datasets or missing data points. Data were extracted using every available age stratum for younger than 5 years. Risk of bias assessments of included studies were conducted using the Quality Assessment of Diagnostic Accuracy Studies (QUADAS-2)^20^ tool by 2 independent reviewers (L.H. and N.B.) with discrepancies resolved by a third reviewer (P.J.G.).

### Statistical Analysis

Diagnostic accuracy results were extracted as 2 × 2 tables for each study for each SARI case definition and virus, as well as age subgroup where possible. We conducted bivariate random-effects meta-analysis of diagnostic test accuracy using the metadata package in Stata version 18.5 (Stata Corp).^21^ For each case definition–virus combination with at least 4 included studies, we calculated pooled estimates of sensitivity and specificity and their 95% CIs. Pooled estimates were visualized using paired forest plots of sensitivity and specificity and summary receiver operating characteristic (SROC) curves. Between-study heterogeneity was assessed by comparing the relative size of confidence and prediction regions in SROC curves as recommended by the Cochrane Handbook for Systematic Reviews of Diagnostic Test Accuracy.^22^ Conceptually, confidence regions are calculated directly from observed SEs of the pooled sensitivity and specificity values of included studies, while prediction regions further consider between-study heterogeneity and reflect the region of sensitivity and specificity estimates in which future or unidentified study would be expected. Closely overlapping confidence and prediction regions indicate low between-study heterogeneity and prediction regions larger than confidence regions indicates greater between-study heterogeneity. Heterogeneity was further summarized with inconsistency index (*I*^2^) statistics calculated using the Zhou and Dendukuri method.^23^ *I*^2^ values were interpreted as low (<50%), moderate (50%-75%), or high (>75%) heterogeneity. Deek funnel plots and asymmetry tests were used to assess for evidence of publication bias,^24^ with a 2-sided *P* < .05 indicating statistical significance. We also conducted age-specific analyses by generating forest plots and SROC curves among studies from which data on children younger than 1 year and 1 year or older could be extracted. Meta-regression was conducted to explore the contribution of viral prevalence on heterogeneity of sensitivity and specificity statistics. All pooled analyses were performed using Stata version 18.5.

## Results

### Description of Included Studies

After duplicate removal, the final search yielded 1144 articles, of which 59 full-text articles were reviewed, and 13 studies^8,9,10,14,16,25,26,27,28,29,30,31,32^ were included (eFigure 1 in Supplement 1). The 13 included observational studies represent pediatric hospitalizations at 65 sites across 8 countries between 2007 and 2023 (Table 1). Sample sizes ranged between 243 and 9969 patients. Included studies were conducted in Canada (2 studies^8,28^), India (2 studies^25,27^), South Africa (2 studies^31,32^), Egypt (1 study^10^), Ghana (1 study^26^), Jordan (1 study^14^), Kenya (2 studies^16,30^), and New Zealand (1 study^9^).

**Table 1.** Characteristics of Included Studies That Evaluated the Diagnostic Accuracy of Case Definitions for SARI in Detecting Viral Infections in Hospitalized Children

Source	Sample size	Country	Surveillance time period	No.of sites	Reference standard	Viruses of interest	Age restriction	Case definitions used
Gupta et al,^27^ 2013	257	India	2009-2011	33	rRT-PCR (NP and OP)	Influenza	<5 y^a^	IMCI pneumonia
Murray et al,^29^ 2013	3116	Kenya	2007-2010	7	rRT-PCR (NP and OP)	Influenza	<5 y^a^	IMCI severe pneumonia
Saha et al,^25^ 2015	505	India	2009-2012	33	rRT-PCR (NP and OP)	RSV	<5 y	WHO 2011 SARI
Jones et al,^26^ 2016	540	Ghana	2010-2013	3	rRT-PCR (NP and OP)	Influenza	<5 y	Combined general SARI definition
Makokha et al,^30^ 2016	3833	Kenya	2009-2013	1	rRT-PCR (NP and OP)	Influenza	<5 y^a^	WHO 2014 SARI; IMCI severe pneumonia
Nyawanda et al,^16^ 2016	3810	Kenya	2009-2013	1	rRT-PCR (NP and OP)	RSV	<5 y	WHO 2014 SARI; IMCI severe pneumonia
Amini et al,^8^ 2017	243	Canada	2012-2013; 2014-2015	4	rRT-PCR (NP)	Influenza	<5 y	WHO 2014 SARI
Ngobeni et al,^31^ 2019	2257	South Africa	2011-2015	2	rRT-PCR (NP)	Influenza	<5 y	WHO 2014 SARI; IMCI severe pneumonia
Rha et al,^32^ 2019	9969	South Africa	2009-2014	5	rRT-PCR (NP)	RSV	<5 y	WHO 2014 SARI
Klink et al,^14^ 2020	3164	Jordan	2010-2013	1	rRT-PCR (NP and OP)	Influenza, RSV, and 4 other viruses	<2 y	WHO 2014 SARI
Rowlinson et al,^10^ 2021	2193	Egypt	2009-2012	3	rRT-PCR (NP and OP)	Influenza	<5 y^a^	WHO 2014 SARI; WHO 2011 SARI; IMCI severe pneumonia
Davis et al,^9^ 2022	3224	New Zealand	2012-2016	4	rRT-PCR (NP and OP)	Influenza and RSV	<5 y^a^	WHO 2014 SARI
Gill et al,^28^ 2025	1485	Canada	2022-2023	2	rRT-PCR (NP and OP)	Influenza, RSV, and 7 other viruses	<5 y	WHO 2014 SARI

Abbreviations: IMCI, Integrated Management of Childhood Illness; NP, nasopharyngeal sample; OP, oropharyngeal sample; rRT-PCR, real-time reverse transcription polymerase chain reaction; RSV, respiratory syncytial virus; SARI, severe acute respiratory infection; WHO, World Health Organization.

^a^ Oldest extracted stratum of nonpediatric specific study.

The WHO 2014 SARI case definition was the most common definition evaluated (9 studies^8,9,10,14,16,28,30,31,32^), alongside the IMCI severe pneumonia definition (5 studies^10,16,29,30,31^), the WHO 2011 SARI definition (2 studies^10,25^), a general IMCI pneumonia definition (1 study^27^), and a combined definition consisting of 5 other definitions (1 study^26^). All studies used rRT-PCR as a reference standard. Viral pathogens included influenza (10 studies^8,9,10,14,26,27,28,29,30,31^) and RSV (6 studies^9,14,16,25,28,32^). Seven studies^8,10,26,27,29,30,31^ evaluated diagnostic accuracy against only influenza, 3 studies^16,25,32^ evaluated against only RSV, and 1 study^9^ evaluated against both influenza and RSV. In addition to influenza and RSV, 2 studies^14,28^ also included parainfluenza, enterovirus or rhinovirus, adenovirus, and human metapneumovirus, of which 1 study^28^ further included SARS-CoV-2, seasonal coronavirus, and bocavirus. Six studies^14,16,25,28,31,32^ were pediatric-specific, of which 1 study^14^ only included patients younger than 2 years.

Table 2 summarizes the diagnostic accuracy of the evaluated case definitions across the included studies. Among studies evaluating influenza, the proportion of patients with influenza-positive status ranged from 3.8% to 20.2% with a median (IQR) of 6.8% (6.1%-8.5%). Among studies evaluating RSV, the proportion of patients with RSV-positive status ranged from 12.3% to 44.1% with a median (IQR) of 34.4% (19.0%-41.7%). eTable 1 in Supplement 1 summarizes the QUADAS-2 risk of bias assessment results. All studies, except Jones et al,^26^ displayed low risk of bias across all domains.

**Table 2.** Diagnostic Accuracy of Case Definitions for SARI in Detecting Viral Infections in Hospitalized Children

Source	Viruses of interest	Virus-positive patients, No. (%)	Case definitions used	SARI positive patients, No. (%)	Sensitivity, %	Specificity, %
Gupta et al,^27^ 2013	Influenza	18/257 (7.0)	IMCI pneumonia	139/257 (54.1)	67.0	47.0
Murray et al,^29^ 2013	Influenza	211/3116 (6.8)	IMCI severe pneumonia	2432/3116 (78.1)	71.1	21.4
Saha et al,^25^ 2015	RSV	82/505 (16.2)	WHO 2011 SARI	123/505 (24.4)	51.2	80.9
Jones et al,^26^ 2016	Influenza	36/540 (6.7)	General combined SARI	330/540 (61.1)	77.8	40.1
Makokha et al,^30^ 2016	Influenza	229/3833 (6.0)	WHO 2014 SARI	2985/3833 (78.0)	84.3	22.5
			IMCI severe pneumonia	2518/3833 (65.7)	69.4	34.5
Nyawanda et al,^16^ 2016	RSV	470/3810 (12.3)	WHO 2014 SARI	2968/3810 (77.9)	82.8	22.8
			IMCI Severe pneumonia	1955/3810 (51.3)	50.2	48.5
Amini et al,^8^ 2017	Influenza	49/243 (20.2)	WHO 2014 SARI	178/243 (73.3)	65.3	24.7
Ngobeni et al,^31^ 2019	Influenza	120/2257 (5.3)	WHO 2014 SARI	1254/2257 (55.6)	66.0	45.0
			IMCI severe pneumonia	1532/2257 (67.9)	66.0	32.0
Rha et al,^32^ 2019	RSV	2723/9969 (27.3)	WHO 2014 SARI	6104/9969 (61.2)	63.6	39.7
Klink et al,^14^ 2020	Influenza	119/3164 (3.8)	WHO 2014 SARI	1261/3164 (39.9)	53.0	60.7
	RSV	1396/3164 (44.1)	WHO 2014 SARI	1261/3164 (39.9)	48.6	66.9
Rowlinson et al,^10^ 2021	Influenza	198/2193 (9.0)	WHO 2014 SARI	1933/2193 (88.1)	80.8	11.1
			WHO 2011 SARI	1772/2193 (80.8)	68.7	18.0
			IMCI severe pneumonia	1270/2193 (57.9 )	38.9	40.9
Davis et al,^9^ 2022	Influenza	385/3224 (11.9)	WHO 2014 SARI	2596/3224 (79.6)	90.9	22.0
	RSV	1304/3143 (41.5)	WHO 2014 SARI	2498/3143 (79.5)	86.6	25.6
Gill et al,^28^ 2025	Influenza	94/1480 (6.4)	WHO 2014 SARI	873/1480 (59.0)	71.3	41.9
	RSV	621/1485 (41.8)	WHO 2014 SARI	873/1485 (58.8)	61.0	42.8

Abbreviations: IMCI, Integrated Management of Childhood Illness; RSV, respiratory syncytial virus; SARI, severe acute respiratory infection; WHO, World Health Organization.

### Diagnostic Accuracy of Case Definitions

**Figure 1.**
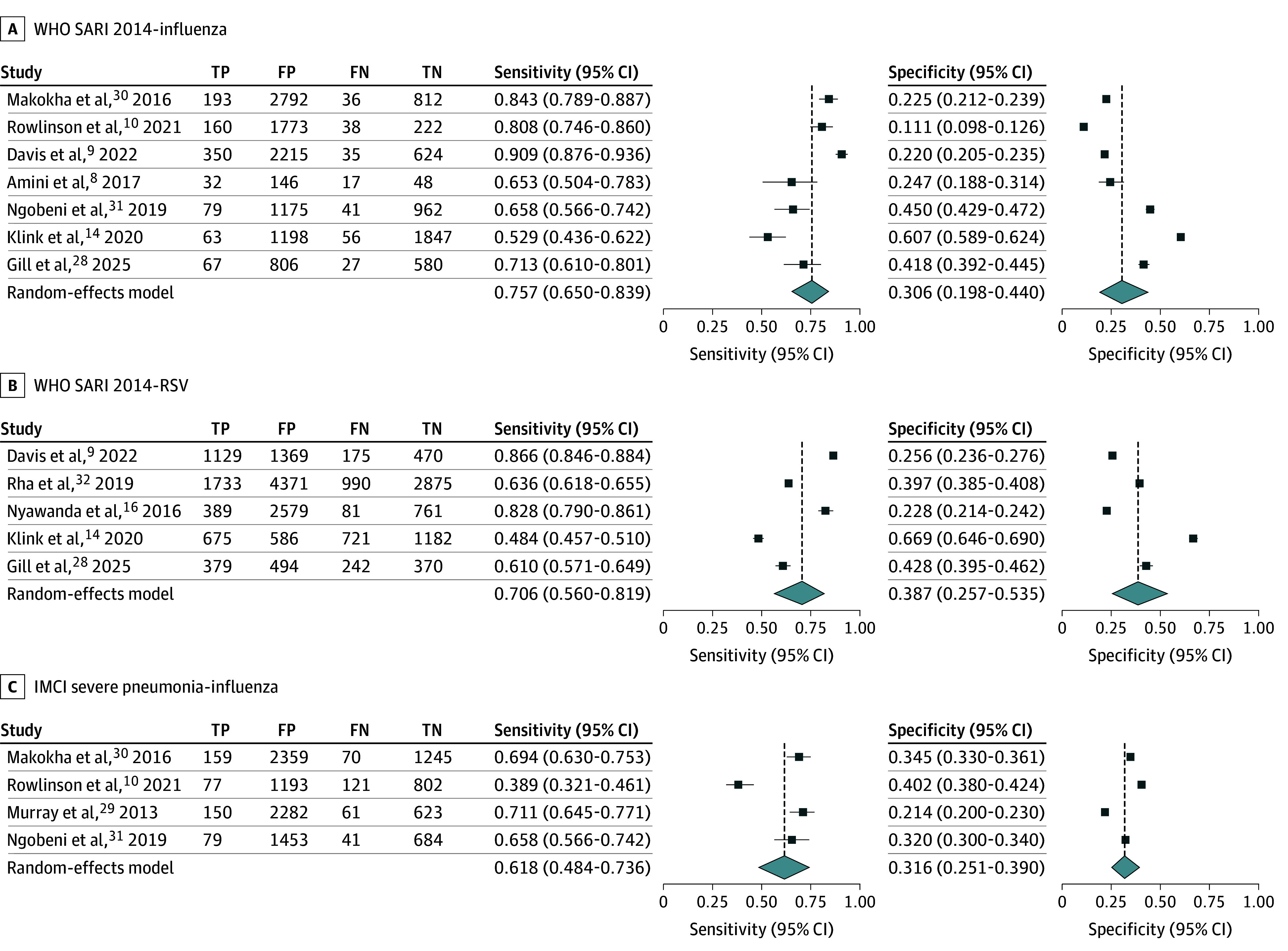
Forest Plots of Diagnostic Accuracy Pooled Analysis FN indicates false negative; FP, false positive; IMCI, Integrated Management for Childhood Illness; SARI, severe acute respiratory infection; TN, true negative; TP, true positive; WHO, World Health Organization.

**Figure 2.**
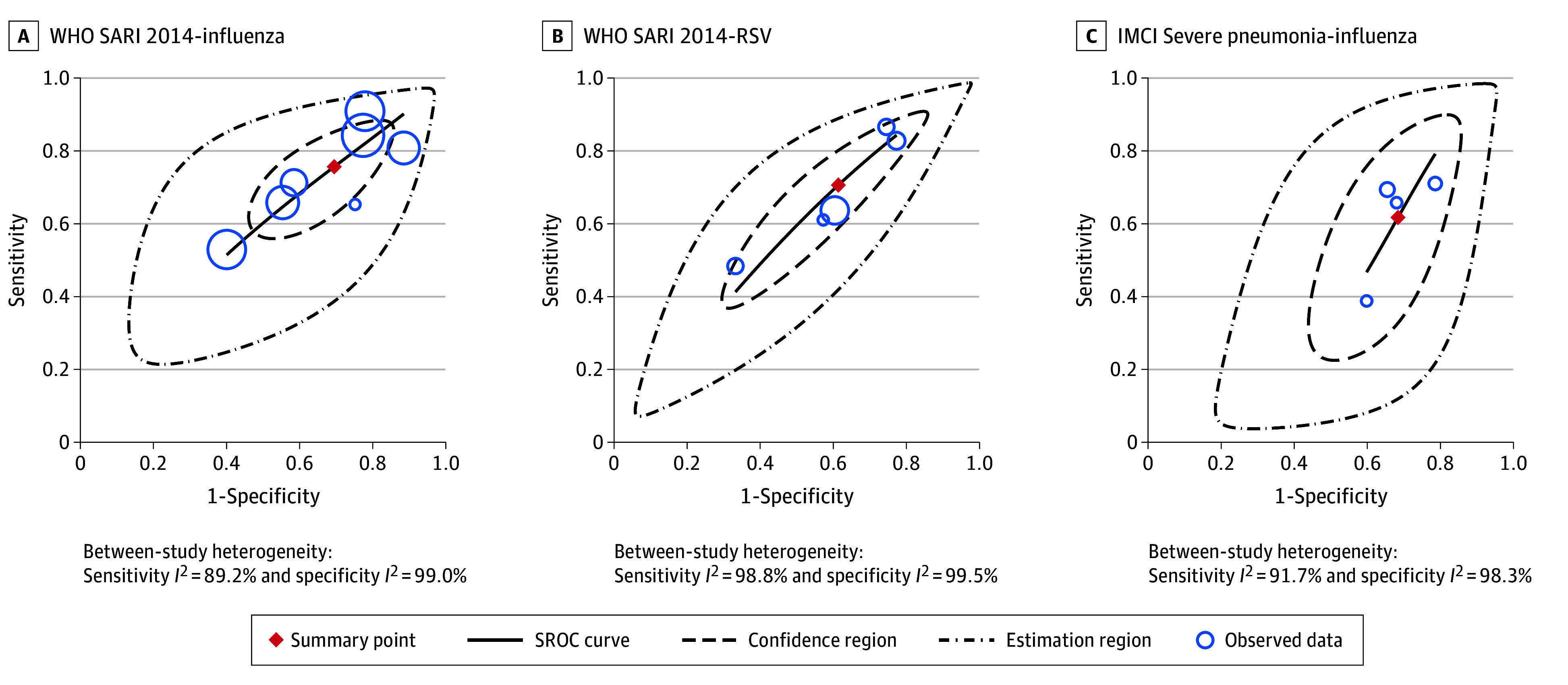
Summary Receiver Operating Characteristic (SROC) Plots of Diagnostic Accuracy Pooled Analysis IMCI indicates Integrated Management for Childhood Illness; SARI, severe acute respiratory infection; WHO, World Health Organization.

Pooled analyses of sensitivity and specificity were feasible for the WHO 2014 SARI definition against both influenza and RSV and for the IMCI severe pneumonia definition against influenza. Figure 1 displays forest plots for pooled estimates. Figure 2 displays SROC curves, confidence regions, and prediction regions for pooled estimates.

Meta-analysis of the WHO 2014 SARI definition against influenza in all age groups (7 studies^8,9,10,14,28,30,31^) yielded pooled estimates of 75.7% (95% CI, 65.0%-83.9%) for sensitivity and 30.6% (95% CI, 19.8%-44.0%) for specificity, with high between-study heterogeneity (sensitivity: *I*^2^ = 89.2%; specificity: *I*^2^ = 99.0%). Meta-analysis of the WHO 2014 SARI definition against RSV in all age groups (5 studies^9,14,16,28,32^) yielded pooled estimates of 70.6% (95% CI, 56.9%-81.9%) for sensitivity and 38.7% (95% CI, 25.7%-53.5%) for specificity, with high between-study heterogeneity (sensitivity: *I*^2^ = 98.8%; specificity: *I*^2^ = 99.5%).

The IMCI severe pneumonia definition was evaluated in 4 studies^10,29,30,31^ against influenza; meta-analysis yielded pooled estimates of 61.8% (95% CI, 48.4%-73.6%) for sensitivity and 31.6% (95% CI, 25.1%-39.0%) for specificity, with high between-study heterogeneity (sensitivity: *I*^2^ = 91.7%; specificity: *I*^2^ = 98.3%).

The high heterogeneity in all 3 pooled combinations was also supported with prediction regions consistently larger than confidence regions. Virus prevalence contributed significantly to between-study heterogeneity, being positively associated with sensitivity statistics and negatively associated with specificity statistics (eTable 2 in Supplement 1). We did not observe asymmetry in the Deek funnel plot analysis for any case definition–virus combination, suggesting a lack of publication bias (eFigure 2 in Supplement 1). The single study examining the IMCI severe pneumonia definition against RSV, Nyawanda et al,^16^ reported a sensitivity of 50.2% (95% CI, 45.7%-54.7%) and specificity of 48.5% (95% CI, 46.8%-50.2%).

The WHO 2011 SARI definition was evaluated once against influenza and once against RSV. Against influenza, Rowlinson et al^10^ reported that the 2011 definition yielded a sensitivity of 68.7% (95% CI, 61.7%-75.1%) and a specificity of 18.0% (95% CI, 16.3%-19.8%). Against RSV, Saha et al^25^ reported that the 2011 definition yielded a sensitivity of 51.2% (95% CI, 39.9%-62.4%) and a specificity of 80.9% (95% CI, 76.8%-84.5%).

Jones et al^26^ combined 5 established definitions: the WHO 2011 SARI, the IMCI severe and moderate pneumonia definitions, the 2006 Pan American Health Organization Adult SARI definition, and the Center for Disease Control and Prevention International Emerging Infections Program pneumonia definition. This expanded definition was evaluated against influenza and reported a sensitivity of 77.8% and specificity of 40.1%. Finally, Gupta et al^27^ evaluated a general IMCI definition which combined patients meeting either the pneumonia or severe or very severe pneumonia criteria against influenza, yielding a sensitivity of 67.0% and specificity of 47.0%. Only 2 studies reported diagnostic accuracy against viruses beyond influenza and RSV, Klink et al^14^ and Gill et al,^28^ both of which evaluated the WHO 2014 SARI definition (eTable 3 in Supplement 1).

### Age Subgrouping

Six studies^8,9,14,16,28,32^ reported diagnostic accuracy among more granular age subgroups beyond the larger younger than 5 years cohort or younger than 2 years cohort. The most common age subgroups included younger than 3 months (4 studies^14,27,28,32^) and younger than 1 year (6 studies^8,9,14,16,28,32^). eTable 4 in Supplement 1 summarizes diagnostic accuracy results of extracted age subgroups. Against influenza, 4 studies^8,9,14,28^ evaluated the WHO 2014 SARI definition in patients younger than 1 year, yielding sensitivities ranging between 44.4% to 87.4% and specificities ranging between 25.6% to 64.3%. In patients younger than 3 months, 3 studies^9,14,28^ evaluated the WHO 2014 SARI definition against influenza, yielding sensitives ranging between 15.9% to 74.1% and specificities between 41.6% to 80.6%.

Against RSV, 5 studies^9,14,16,28,32^ evaluated the WHO 2014 SARI definition in patients younger than 1 year, yielding sensitivities ranging between 44.3% to 81.5% and specificities ranging between 21.1% to 71.2%. In patients younger than 3 months, 4 studies^9,14,28,32^ evaluated the 2014 definition against RSV, yielding sensitivities ranging between 23.9% to 62.4%, and specificities between 43.3% to 85.1%. Against both influenza and RSV, every study which stratified by age saw a successive reduction in sensitivity and increase in specificity between the 1 year or older, younger than 1 year, and younger than 3 months subgroups.

**Figure 3.**
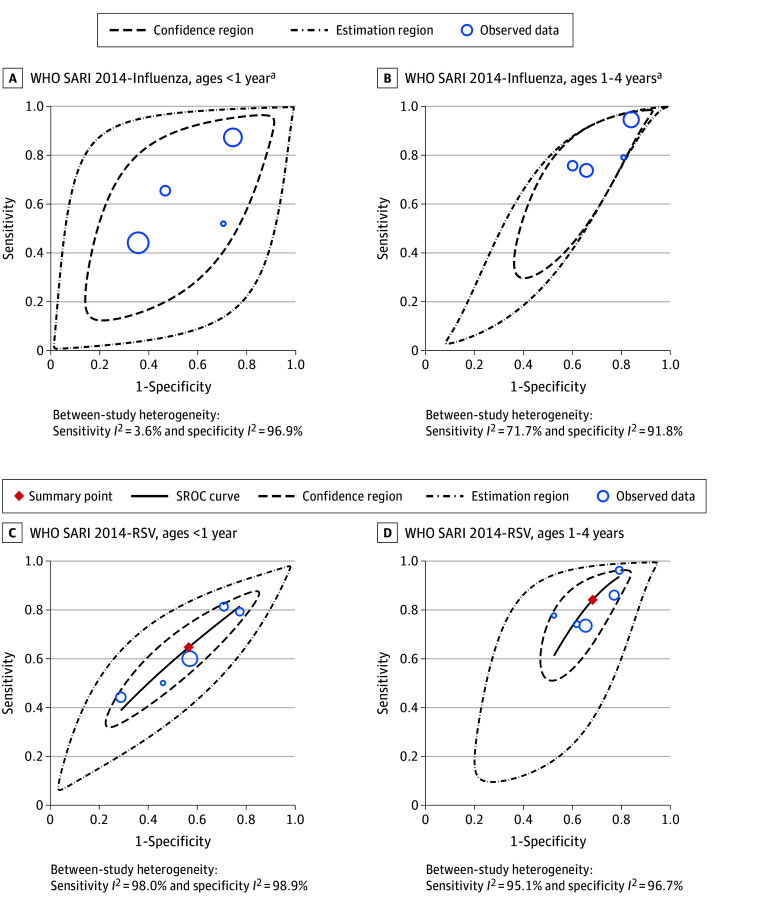
Summary Receiver Operating Characteristic (SROC) Plots of Age Subgrouped Pooled Analysis IMCI indicates Integrated Management for Childhood Illness; RSV, respiratory syncytial virus; SARI, severe acute respiratory infection; WHO, World Health Organization. ^a^Summary points and SROC curves of the WHO SARI 2014 definition for influenza are not presented given excessive heterogeneity observed among ages younger than 1 year.

Pooled analysis was feasible for the WHO 2014 SARI definition against RSV at the younger than 1 year and 1 year or older subgroups (5 studies^9,14,16,28,32^). This resulted in a pooled sensitivity of 64.8% (95% CI, 50.0%-77.3%) and specificity of 43.4% (95% CI, 28.2%-60.0%) for younger than 1 year and a pooled sensitivity of 84.3% (95% CI, 72.0%-91.8%) and specificity of 31.7% (95% CI, 23.8%-40.9%) for 1 year or older. Age-specific pooled analysis of influenza was not possible given the excessive between-study heterogeneity upon SROC analysis (4 studies^8,9,14,28^). Figure 3 displays SROC curves, confidence regions, and prediction regions for age-subgrouped pooled estimates. eFigure 3 in Supplement 1 displays forest plots of the age-subgrouped pooled estimates.

## Discussion

This systematic review and meta-analysis evaluated and compared the diagnostic accuracy of established SARI case definitions for viral acute respiratory tract infections among children and youths. The most studied case definition was the 2014 WHO SARI definition. Further, SARI case definitions were mostly evaluated in studies against influenza, with several studies evaluating against RSV, and only 2 studies^14,28^ evaluating against other viruses. The included studies evaluated case definitions across a range of countries of various regions and wealth categories, most prior to the COVID-19 pandemic, with a focus almost entirely on the younger than 5 years age group. The diagnostic accuracy of the WHO SARI case definition varied. Our study highlights the limited understanding of the diagnostic accuracy of the SARI case definition in pediatrics. We further highlight the near complete absence of viruses other than influenza and RSV, notably SARS-CoV-2.

Against influenza, the diagnostic performance of the WHO 2014 SARI definition varied greatly, with wide ranges in sensitivity and specificity. Overall, the pooled analysis of 7 studies^8,9,10,14,28,30,31^ yielded a sensitivity of 75.7% and specificity of 30.6% in pediatric cohorts against influenza; this is moderately less sensitive and more specific than past evaluations of the 2014 definition against influenza in adult populations, which ranged between 78.8% to 91.9% in sensitivity and 18.0% to 32.5% in specificity. Against RSV, the WHO 2014 SARI definition yielded similar variation to influenza, with equally high between-study heterogeneity. Overall, the pooled analysis of 5 studies^9,14,16,28,32^ yielded a sensitivity of 70.6% and specificity of 38.7%, which is notably less sensitive and more specific than the findings of a systematic review on the 2014 definition against RSV in adults, which reported sensitivities ranging between 78.8% to 89% and specificities ranging between 6.6% to 27.7%.^12,13^ These findings reaffirm that the 2014 WHO definition favored for viral surveillance is less sensitive in pediatric populations compared with adults.

The difference in diagnostic accuracy of the 2014 SARI definition between children and adults can likely be attributed to differences in symptom presentation, particularly the fever criterion, which is notably less common in young children.^14,28,33^ In fact, afebrile pneumonitis is well documented in pediatrics, with some studies reporting that 70% to 80% of young infants with viral pneumonia will present without fever.^34,35^ Studies which evaluated a modified SARI definition without the requirement of fever reported greatly increased sensitivity with varying decreases in specificity.^14,28,33^ Furthermore, young children exhibit significantly higher rates of viral coinfections,^36^ which may complicate the clinical picture and render symptom-based surveillance definitions less accurate. Children also face a greater diversity in viral infections, which increases the potential reasons for SARI presentation, as opposed to adults.^36,37^

This difference in diagnostic accuracy of the 2014 definition is particularly pronounced in younger age groups. Despite between-study variation, every included study which stratified by age saw a reduction in sensitivity and increase in specificity between the 1 year or older, younger than 1 year, and younger than 3 months subgroups. This trend was further shown by the age-subgrouped pooled analysis against RSV, which found a 19.5 absolute percentage point reduction in sensitivity and an 11.7 absolute percentage point increase in specificity between the 1 year or older cohort and younger than 1 year cohort. These differences suggest the 2014 definition misses a greater number of viral infections in the youngest patients as opposed to older pediatric cohorts.

A key implication of the observed lower sensitivity of SARI case definitions among younger cohorts is the potential for the underestimation of disease burden. SARI sentinel surveillance systems are routinely used to estimate viral respiratory disease burden by calculating the incidence of virus-associated SARI for a given pathogen and extrapolating impact on the larger population,^38,39,40^ per WHO guidelines on this practice.^41^ Our findings confirm that such calculations of incidence among younger cohorts should be adjusted to consider the sensitivity of the case definition applied. The decrease in sensitivity among pediatric populations increases the proportion of false negatives and undermines the validity of key public health conclusions. Because increasing the sensitivity of SARI case definitions may not be feasible, especially in resource-limited regions without the capacity for more laboratory testing, it is crucial that SARI sentinel surveillance accounts for the disparity in diagnostic accuracy between young children and adults when estimating viral burden from viral-associated SARI. It is also possible that the sensitivities of SARI case definitions differ by other factors, such as geographical region, vaccination status, and access to care. While our review primarily noted differences in diagnostic accuracy by age group and pathogen, future investigations should examine other modifiers to provide a more complete picture of SARI sentinel surveillance and identify contexts where published estimates of sensitivity and specificity may not be applicable.

We reaffirm the importance of viral surveillance practices that accurately recognize trends and estimate disease impact in pediatric populations, who are disproportionately affected by respiratory pathogens.^42^ To our knowledge, this study is the first systematic review of diagnostic accuracy of SARI definitions specifically in inpatient pediatric populations. The breadth in geography and time periods of included studies suggests our findings are generalizable to most existing viral surveillance systems.

### Limitations

This review also faces several limitations. First, the study compared published peer reviewed studies, potentially missing public health surveillance and other gray literature reports. We aimed to mitigate this by searching gray literature, which yielded no additional studies. Future SARI case definition research may need a heightened focus on public health database reporting, as opposed to peer reviewed studies. Second, the included studies yielded wide between-study heterogeneity, which is common in meta-analyses of diagnostic test accuracy.^43^ All included studies utilized similar rRT-PCR testing and hospital-based samples, and importantly were not subject to threshold effects as may be the case in meta-analyses of tests with continuous measurements. However, case mix (prevalence) was an important contributor to heterogeneity, suggesting sensitivity and specificity of case definitions may shift in different geographies and seasons. High heterogeneity may further be expected due to the inherent nonspecific symptoms in children compared with adults, as well as the observational designs, but we felt the pooled analysis was appropriate nonetheless given our focus on understanding the relevance of a broad case definition. Third, some meta-analyses were conducted among only 4 or 5 studies, which may decrease the reliability of the resulting statistical inferences. Additionally, most studies focused on seasonal influenza, with some recent studies focusing on RSV. This resulted in limited data on other viruses. Conventional surveillance methods have been found to be inadequate for emerging pandemic threats, such as H5N1 avian influenza, which has not been studied from a SARI perspective.^44^ While sentinel surveillance is primarily designed to monitor trends over time, it can aid in identifying unusual patterns or clusters, complementing other systems such as event-based surveillance and media monitoring. Developing a clearer picture of the performance of existing SARI surveillance infrastructure against other viruses will help health systems prepare for public health challenges.

## Conclusions

This systematic review of 13 studies evaluating the diagnostic accuracy of SARI case definitions in detecting viral infections found that the most common definition, the WHO 2014 SARI definition, yielded moderate to poor sensitivity in pediatric populations against both influenza and RSV. While more specific than adult benchmarks, the reduced sensitivity may lead to a greater proportion of missed cases in surveillance systems that rely on SARI case definitions to estimate respiratory viral disease burden. This trend was particularly pronounced in the younger than 1 year subgroup. Alternative case definitions, such as the 2011 WHO SARI definition or the IMCI severe pneumonia definition, were also less sensitive than the 2014 definition, regardless of virus or age cohort. SARI sentinel surveillance systems which account for pediatric differences are needed to enhance pandemic readiness.

## Notes

Supplement 1. **eMethods.** Updated Search Strategy **eFigure 1.** PRISMA Flowchart of Study Screening **eTable 1.** Risk of Bias Assessment by QUADAS-2 of Included Studies **eTable 2.** Meta-Regression Associations Between Prevalence and Test Accuracy (Sensitivity and Specificity) Statistics **eFigure 2.** Publication Bias Plots of Diagnostic Accuracy Pooled Analysis **eTable 3.** Diagnostic Accuracy of WHO 2014 SARI Definition Against Other Viruses **eTable 4.** Age Sub-Grouped Diagnostic Accuracy of SARI Case Definitions in Included Studies **eFigure 3.** Forest Plots of Age Sub-Grouped Analysis

Supplement 2.
Data Sharing Statement
