# Supplementary material for: Diagnostic Accuracy of Severe Acute Respiratory Infection Definitions in Hospitalized Children: A Systematic Review and Meta-Analysis
Source: JAMA Netw Open. 2025 Dec 18;8(12):e2550298. doi: 10.1001/jamanetworkopen.2025.50298 (PMC12715654; doi:10.1001/jamanetworkopen.2025.50298)
Supplement: Supplement 1. — eMethods. Updated Search Strategy eFigure 1. PRISMA Flowchart of Study Screening eTable 1. Risk of Bias Assessment by QUADAS-2 of Included Studies eTable 2. Meta-Regression Associations Between Prevalence and Test Accuracy (Sensitivity and Specificity) Statistics eFigure 2. Publication Bias Plots of Diagnostic Accuracy Pooled Analysis eTable 3. Diagnostic Accuracy of WHO 2014 SARI Definition Against Other Viruses eTable 4. Age Sub-Grouped Diagnostic Accuracy of SARI Case Definitions in Included Studies eFigure 3. Forest Plots of Age Sub-Grouped Analysis [file jamanetwopen-e2550298-s001.pdf]

Supplemental Online Content

Hersi L, Kant T, Kaziev CL, et al. Diagnostic accuracy of severe acute respiratory infections definitions in hospitalized children. *JAMA Netw. Open*. 2025;8(12):e2550298. doi:10.1001/jamanetworkopen.2025.50298

eMethods. Updated Search Strategy

eFigure 1. PRISMA Flowchart of Study Screening

eTable 1. Risk of Bias Assessment by QUADAS-2 of Included Studies

eTable 2. Meta-Regression Associations Between Prevalence and Test Accuracy (Sensitivity and Specificity) Statistics

eFigure 2. Publication Bias Plots of Diagnostic Accuracy Pooled Analysis

eTable 3. Diagnostic Accuracy of WHO 2014 SARI Definition Against Other Viruses

eTable 4. Age Sub-Grouped Diagnostic Accuracy of SARI Case Definitions in Included Studies

eFigure 3. Forest Plots of Age Sub-Grouped Analysis

This supplemental material has been provided by the authors to give readers additional information about their work.

© 2025 Hersi L et al. *JAMA Network Open*.

eMethods. Updated Search Strategy

Search methods statement
The search was executed in the following bibliographic databases on June 11, 2024: Ovid MEDLINE(R) and Epub Ahead of Print, In-Process, In-Data-Review & Other Non-Indexed Citations and Daily <1946 to June 10, 2024>; Ovid Embase+Embase Classic (1974–2024 week 23); Ovid EBM Reviews Cochrane Central Register of Controlled Trials (May 2024); Elsevier SCOPUS (June 11, 2024); and WHO Global Index Medicus (June 11, 2024). The search strategy consisted of both controlled vocabulary, such as the National Library of Medicine's MeSH (Medical Subject Headings) and Emtree Subject Headings (Embase), and keywords. No date or language limits were applied to the original search. The search was updated on March 31, 2025 using the same parameters, except date limits were applied from the date of the previous search to the current date. See Appendix [To be added] for the detailed search strategy.
Search strategy:f
Ovid MEDLINE(R) Epub Ahead of Print and In-Process, In-Data-Review & Other Non-Indexed Citations and Daily 1946 to March 28, 2025

Ovid MEDLINE(R) Epub Ahead of Print and In-Process, In-Data-Review & Other Non-Indexed Citations and Daily 1946 to March 28, 2025

#	Searches	Results
1	"Sensitivity and Specificity"/ or Diagnosis/ or "Population Surveillance"/mt or "Sentinel Surveillance"/ or ((Sentinel* or biosurveillance or "bio-surveillance") and ("health event?" or site or sites or illness or system? or surveill* or diagnos* or "positive predictive value" or sensitivity or specificity)).mp.	438686
2	"Multiplex Polymerase Chain Reaction"/ or ("Multiplex Polymerase Chain Reaction*" or "Multiplex PCR" or MPCR or "Triplex Polymerase Chain Reaction*" or "Multiplex Ligation-Dependent Probe Amplification").tw,kf.	22198
3	1 or 2	457865
4	(("Severe Acute Respiratory Infect*" or SARI or sentinel) and ("case definition*" or case or cases or incidence or epidemiology or "test* characteristic?" or "clinical* characteristic?" or evaluat*)).mp.	27862
5	3 and 4	20683
6	"Respiratory Tract Infections"/ or ("Respiratory Tract Infection?" or "Respiratory System Infection?" or "Respiratory Infection?" or "Upper Respiratory Tract Infection?" or "respiratory virus*" or "triple-demic*" or "tripledemic*" or "viral surge?").mp.	90500
7	(COVID-19/ or exp COVID-19 Testing/ or COVID-19 Vaccines/ or SARS-CoV-2/ or (coronavirus/ or betacoronavirus/ or coronavirus infections/)) and (disease outbreaks/ or epidemics/ or pandemics/)	133484
8	(nCoV* or 2019nCoV or 19nCoV or COVID19* or COVID or SARS-COV-2 or SARSCOV-2 or SARS-COV2 or SARSCOV2 or SARS coronavirus 2 or Severe Acute Respiratory Syndrome Coronavirus 2 or Severe Acute Respiratory Syndrome Corona Virus 2).ti,ab,kf,nm,ot,ox,rx,px.	451465
9	((new or novel or "19" or "2019" or Wuhan or Hubei or China or Chinese) adj3 (coronavirus* or corona virus* or betacoronavirus* or CoV or HCoV)).ti,ab,kf,ot.	108376
10	(longCOVID* or postCOVID* or postcoronavirus* or postSARS*).ti,ab,kf,ot.	126

© 2025 Hersi L et al. *JAMA Network Open*.

11	((coronavirus* or corona virus* or betacoronavirus*) adj3 (pandemic* or epidemic* or outbreak* or crisis)).ti,ab,kf,ot.	18263
12	((Wuhan or Hubei) adj5 pneumonia).ti,ab,kf,ot.	437
13	"Middle East Respiratory Syndrome Coronavirus"/ or ("Middle East Respiratory Syndrome Coronavirus" or "MERS-CoV" or "MERS Virus" or MERS or "Middle East respiratory syndrome-related coronavirus" or Merbecovirus*).mp.	9548
14	"Respiratory Syncytial Virus Infections"/ or ("Respiratory Syncytial Virus Infection?" or "RSV Infection?").mp.	12158
15	"Influenza, Human"/ or ("Human Influenza?" or ((influenza or flu) adj3 human?) or grippe or influenza?).mp.	163390
16	*Metapneumovirus/ or "human metapneumovirus*".tw,kf.	2561
17	"Adenoviridae Infections"/ or ("Human Adenovirus Infection*" or "Adenovir* Infection?").tw,kf.	8519
18	"Human bocavirus"/ or "Human bocavirus*".tw,kf.	1010
19	"Parainfluenza Virus 1, Human"/ or "Parainfluenza Virus 2, Human"/ or "Parainfluenza Virus 3, Human"/ or "Parainfluenza Virus 4, Human"/ or ("Hemadsorption Virus 2" or "Human parainfluenza virus 1" or "Parainfluenza Virus Type 1" or "Para-Influenza Virus Type 1" or "Croup Associated Virus" or "Acute Laryngotracheobronchitis Virus*" or "Para-Influenza Virus Type 2" or "Acute Laryngotracheo-Bronchitis Virus*" or "Human parainfluenza virus 2" or "Hemadsorption Virus 1" or "Human parainfluenza virus 3" or "Parainfluenza Virus Type 3" or "Para-Influenza Virus Type 3" or "Human parainfluenza virus 4" or "parainfluenza virus type 4" or "para-influenza virus type 4").tw,kf.	4721
20	or/6-19	706128
21	5 and 20	2446
22	exp "Acute disease"/ or Cough/vi, di, et or Fever/vi, di, et or (cough* or fever? or Pyrexia? or (acute adj2 (disease* or condition? or ill* or symptom*))).mp.	616790
23	Hospitalization/ or ((patient? adj3 (hospitali* or admission* or admit* or readmi*)) or (hospital adj2 admi*)).mp.	486930
24	22 or 23	1067332
25	exp adolescent/ or exp child/ or exp infant/ or *pediatrics/ or (infant disease* or childhood disease*).ti,ab,kf. or (adolescen* or babies or baby or boy? or boyhood or girlhood or child* or girl? or infan* or juvenil* or kid? or minors or minors* or neonat* or neo-nat* or newborn* or new-born* or paediatric* or peadiatric* or pediatric* or perinat* or preschool* or puber* or pubescen* or school* or teen* or toddler? or underage? or under-age? or youth*).ti,ab,kf. or (pediatric* or paediatric* or infan* or child* or adolescen* or young).jn,jw. or (pediatric* or paediatric* or infan* or child* or adolescen* or young).in.	6124558
26	24 and 25	290077
27	21 and 26	524
28	remove duplicates from 27	522
29	limit 28 to dt=20240610-20250331	27

© 2025 Hersi L et al. *JAMA Network Open*.

30	limit 28 to ed=20240610-20250331	27
31	limit 28 to ez=20240610-20250331	27
32	or/29-31	29

Ovid Embase Classic+Embase <1947 to 2025 Week 13>

#	**Searches**	**Results**
1	"Sensitivity and Specificity"/ or Diagnosis/ or "Population Surveillance"/ or "Sentinel Surveillance"/ or ((Sentinel* or biosurveillance or "bio-surveillance") and ("health event?" or site or sites or illness or system? or surveill* or diagnos* or "positive predictive value" or sensitivity or specificity)).mp.	2388042
2	"Multiplex Polymerase Chain Reaction"/ or ("Multiplex Polymerase Chain Reaction*" or "Multiplex PCR" or MPCR or "Triplex Polymerase Chain Reaction*" or "Multiplex Ligation-Dependent Probe Amplification").tw,kf.	38304
3	1 or 2	2420192
4	(("Severe Acute Respiratory Infect*" or SARI or sentinel) and ("case definition*" or case or cases or incidence or epidemiology or "test* characteristic?" or "clinical* characteristic?" or evaluat*)).mp.	40794
5	3 and 4	29325
6	"Respiratory Tract Infection"/ or ("Respiratory Tract Infection?" or "Respiratory System Infection?" or "Respiratory Infection?" or "Upper Respiratory Tract Infection?" or "respiratory virus*" or "triple-demic*" or "tripledemic*" or "viral surge?").mp.	179458
7	("coronavirus disease 2019"/ or exp "COVID-19 Testing"/ or "SARS-CoV-2 vaccine"/ or "Severe acute respiratory syndrome coronavirus 2"/ or (Coronavirinae/ or betacoronavirus/ or coronavirus infection/)) and (epidemic/ or pandemic/)	181780
8	(nCoV* or 2019nCoV or 19nCoV or COVID19* or COVID or SARS-COV-2 or SARSCOV-2 or SARS-COV2 or SARSCOV2 or SARS coronavirus 2 or Severe Acute Respiratory Syndrome Coronavirus 2 or Severe Acute Respiratory Syndrome Corona Virus 2).ti,ab,kf,ot,ox,px.	524020
9	((new or novel or "19" or "2019" or Wuhan or Hubei or China or Chinese) adj3 (coronavirus* or corona virus* or betacoronavirus* or CoV or HCoV)).ti,ab,kf,ot.	113425
10	(longCOVID* or postCOVID* or postcoronavirus* or postSARS*).ti,ab,kf,ot.	455
11	((coronavirus* or corona virus* or betacoronavirus*) adj3 (pandemic* or epidemic* or outbreak* or crisis)).ti,ab,kf,ot.	18646
12	((Wuhan or Hubei) adj5 pneumonia).ti,ab,kf,ot.	544
13	"Middle East Respiratory Syndrome Coronavirus"/ or ("Middle East Respiratory Syndrome Coronavirus" or "MERS-CoV" or "MERS Virus" or MERS or "Middle East respiratory syndrome-related coronavirus" or Merbecovirus*).mp.	12754
14	"Respiratory Syncytial Virus Infection"/ or ("Respiratory Syncytial Virus Infection?" or "RSV Infection?").mp.	14436
15	*"Influenza"/ or ("Human Influenza?" or ((influenza or flu) adj3 human?) or grippe or influenza?).mp.	261131

© 2025 Hersi L et al. *JAMA Network Open*.

16	*"Human metapneumovirus"/ or "human metapneumovirus*".tw,kf.	3197
17	"Adenovirus Infection"/ or ("Human Adenovirus Infection*" or "Adenovir* Infection?").tw,kf.	8528
18	"Human bocavirus"/ or "Human bocavirus*".tw,kf.	1457
19	"Human Parainfluenza Virus 1"/ or "Human Parainfluenza Virus 2"/ or "Human Parainfluenza Virus 3"/ or "Human Parainfluenza Virus 4"/ or ("Hemadsorption Virus 2" or "Human parainfluenza virus 1" or "Parainfluenza Virus Type 1" or "Para-Influenza Virus Type 1" or "Croup Associated Virus" or "Acute Laryngotracheobronchitis Virus*" or "Para-Influenza Virus Type 2" or "Acute Laryngotracheo-Bronchitis Virus*" or "Human parainfluenza virus 2" or "Hemadsorption Virus 1" or "Human parainfluenza virus 3" or "Parainfluenza Virus Type 3" or "Para-Influenza Virus Type 3" or "Human parainfluenza virus 4" or "parainfluenza virus type 4" or "para-influenza virus type 4").tw,kf.	7292
20	or/6-19	949835
21	5 and 20	2272
22	"Acute disease"/ or Coughing/di, et or Fever/di, et or (cough* or fever? or Pyrexia? or (acute adj2 (disease* or condition? or ill* or symptom*))).mp.	957342
23	"child hospitalization"/ or Hospitalization/ or ((patient? adj3 (hospitali* or admission* or admit* or readmi*)) or (hospital adj2 admi*)).mp.	1259844
24	22 or 23	2107373
25	exp adolescent/ or exp child/ or exp infant/ or *pediatrics/ or (infant disease* or childhood disease*).ti,ab,kf. or (adolescen* or babies or baby or boy? or boyhood or girlhood or child* or girl? or infan* or juvenil* or kid? or minors or minors* or neonat* or neo-nat* or newborn* or new-born* or paediatric* or peadiatric* or pediatric* or perinat* or preschool* or puber* or pubescen* or school* or teen* or toddler? or underage? or under-age? or youth*).ti,ab,kf. or (pediatric* or paediatric* or infan* or child* or adolescen* or young).jn,jx. or (pediatric* or paediatric* or infan* or child* or adolescen* or young).in.	7383447
26	24 and 25	517497
27	21 and 26	533
28	remove duplicates from 27	529
29	limit 28 to dc=20240610-20250331	45

Ovid EBM Reviews - Cochrane Central Register of Controlled Trials – February 2025

#	Searches	Results
1	"Sensitivity and Specificity"/ or Diagnosis/ or "Population Surveillance"/mt or "Sentinel Surveillance"/ or ((Sentinel* or biosurveillance or "bio-surveillance") and ("health event?" or site or sites or illness or system? or surveill* or diagnos* or "positive predictive value" or sensitivity or specificity)).mp.	12738
2	"Multiplex Polymerase Chain Reaction"/ or ("Multiplex Polymerase Chain Reaction*" or "Multiplex PCR" or MPCR or "Triplex Polymerase Chain Reaction*" or "Multiplex Ligation-Dependent Probe Amplification").tw,kf.	319
3	1 or 2	13042

© 2025 Hersi L et al. *JAMA Network Open*.

4	(("Severe Acute Respiratory Infect*" or SARI or sentinel) and ("case definition*" or case or cases or incidence or epidemiology or "test* characteristic?" or "clinical* characteristic?" or evaluat*)).mp.	1881
5	3 and 4	969
6	"Respiratory Tract Infections"/ or ("Respiratory Tract Infection?" or "Respiratory System Infection?" or "Respiratory Infection?" or "Upper Respiratory Tract Infection?" or "respiratory virus*" or "triple-demic*" or "tripledemic*" or "viral surge?").mp.	13293
7	(COVID-19/ or exp COVID-19 Testing/ or COVID-19 Vaccines/ or SARS-CoV-2/ or (coronavirus/ or betacoronavirus/ or coronavirus infections/)) and (disease outbreaks/ or epidemics/ or pandemics/)	1854
8	[(nCoV* or 2019nCoV or 19nCoV or COVID19* or COVID or SARS-COV-2 or SARSCOV-2 or SARS-COV2 or SARSCOV2 or SARS coronavirus 2 or Severe Acute Respiratory Syndrome Coronavirus 2 or Severe Acute Respiratory Syndrome Corona Virus 2).ti,ab,kf,nm,ot,ox,rx,px.]	0
9	((new or novel or "19" or "2019" or Wuhan or Hubei or China or Chinese) adj3 (coronavirus* or corona virus* or betacoronavirus* or CoV or HCoV)).ti,ab,kf,ot.	4674
10	(longCOVID* or postCOVID* or postcoronavirus* or postSARS*).ti,ab,kf,ot.	44
11	((coronavirus* or corona virus* or betacoronavirus*) adj3 (pandemic* or epidemic* or outbreak* or crisis)).ti,ab,kf,ot.	457
12	((Wuhan or Hubei) adj5 pneumonia).ti,ab,kf,ot.	29
13	"Middle East Respiratory Syndrome Coronavirus"/ or ("Middle East Respiratory Syndrome Coronavirus" or "MERS-CoV" or "MERS Virus" or MERS or "Middle East respiratory syndrome-related coronavirus" or Merbecovirus*).mp.	177
14	"Respiratory Syncytial Virus Infections"/ or ("Respiratory Syncytial Virus Infection?" or "RSV Infection?").mp.	855
15	"Influenza, Human"/ or ("Human Influenza?" or ((influenza or flu) adj3 human?) or grippe or influenza?).mp.	11111
16	*Metapneumovirus/ or "human metapneumovirus*".tw,kf.	72
17	"Adenoviridae Infections"/ or ("Human Adenovirus Infection*" or "Adenovir* Infection?").tw,kf.	122
18	"Human bocavirus"/ or "Human bocavirus*".tw,kf.	15
19	"Parainfluenza Virus 1, Human"/ or "Parainfluenza Virus 2, Human"/ or "Parainfluenza Virus 3, Human"/ or "Parainfluenza Virus 4, Human"/ or ("Hemadsorption Virus 2" or "Human parainfluenza virus 1" or "Parainfluenza Virus Type 1" or "Para-Influenza Virus Type 1" or "Croup Associated Virus" or "Acute Laryngotracheobronchitis Virus*" or "Para-Influenza Virus Type 2" or "Acute Laryngotracheo-Bronchitis Virus*" or "Human parainfluenza virus 2" or "Hemadsorption Virus 1" or "Human parainfluenza virus 3" or "Parainfluenza Virus Type 3" or "Para-Influenza Virus Type 3" or "Human parainfluenza virus 4" or "parainfluenza virus type 4" or "para-influenza virus type 4").tw,kf.	44
20	or/6-19	29689
21	5 and 20	22

© 2025 Hersi L et al. *JAMA Network Open*.

22	exp "Acute disease"/ or Cough/vi, di, et or Fever/vi, di, et or (cough* or fever? or Pyrexia? or (acute adj2 (disease* or condition? or ill* or symptom*))).mp.	63049
23	Hospitalization/ or ((patient? adj3 (hospitali* or admission* or admit* or readmi*)) or (hospital adj2 admi*)).mp.	52744
24	22 or 23	111005
25	exp adolescent/ or exp child/ or exp infant/ or *pediatrics/ or (infant disease* or childhood disease*).ti,ab,kf. or (adolescen* or babies or baby or boy? or boyhood or girlhood or child* or girl? or infan* or juvenil* or kid? or minors or minors* or neonat* or neo-nat* or newborn* or new-born* or paediatric* or peadiatric* or pediatric* or perinat* or preschool* or puber* or pubescen* or school* or teen* or toddler? or underage? or under-age? or youth*).ti,ab,kf. or (pediatric* or paediatric* or infan* or child* or adolescen* or young).jn,jw. or (pediatric* or paediatric* or infan* or child* or adolescen* or young).in.	392416
26	24 and 25	25685
27	21 and 26	3
28	remove duplicates from 27	3
29	limit 28 to yr="2024 -Current"	0

Elsevier SCOPUS – March 31, 2025

N=39

((TITLE-ABS-KEY ("infant disease*" OR "childhood disease*" OR adolescen* OR babies OR baby OR boy? OR boyhood OR girlhood OR child* OR girl? OR infan* OR juvenil* OR kid? OR minors OR minors* OR neonat* OR neo-nat* OR newborn* OR new-born* OR paediatric* OR peadiatric* OR pediatric* OR perinat* OR preschool* OR puber* OR pubescen* OR school* OR teen* OR toddler? OR underage? OR under-age? OR youth*)) AND ((TITLE-ABS-KEY ((patient? W/3 (hospitali* OR admission* OR admit* OR readmi*)) OR (hospital W/2 admi*))) OR (TITLE-ABS-KEY (cough* OR fever? OR pyrexia? OR (acute W/2 (disease* OR condition? OR ill* OR symptom*)))))) AND (((TITLE-ABS-KEY ("Respiratory Tract Infection?" OR "Respiratory System Infection?" OR "Respiratory Infection?" OR "Upper Respiratory Tract Infection?" OR "respiratory virus*" OR "triple-demic*" OR "tripledemic*" OR "viral surge?")) OR (TITLE-ABS-KEY (ncov* OR 2019ncov OR 19ncov OR covid19* OR covid OR sars-cov-2 OR sarscov-2 OR sars-cov2 OR sarscov2 OR "SARS coronavirus 2" OR "Severe Acute Respiratory Syndrome Coronavirus 2" OR "Severe Acute Respiratory Syndrome Corona Virus 2")) OR (TITLE-ABS-KEY ((new OR novel OR "19" OR "2019" OR wuhan OR hubei OR china OR chinese) W/3 (coronavirus* OR "corona virus*" OR betacoronavirus* OR cov OR hcov))) OR (TITLE-ABS-KEY (longcovid* OR postcovid* OR postcoronavirus* OR postsars*)) OR (TITLE-ABS-KEY ((coronavirus* OR "corona virus*" OR betacoronavirus*) W/3 (pandemic* OR epidemic* OR outbreak* OR crisis))) OR (TITLE-ABS-KEY ((wuhan OR hubei) W/5 pneumonia)) OR (TITLE-ABS-KEY ("Middle East Respiratory Syndrome Coronavirus" OR "MERS-CoV" OR "MERS Virus" OR mers OR "Middle East respiratory syndrome-related coronavirus" OR merbecovirus*)) OR (TITLE-ABS-KEY ("Respiratory Syncytial Virus Infection?" OR "RSV Infection?")) OR (TITLE-ABS-KEY ("Human Influenza?" OR ((influenza OR flu) W/3 human?) OR grippe OR influenza?)) OR (TITLE-ABS-KEY ("human metapneumovirus*")) OR (TITLE-ABS-KEY ("Human Adenovirus Infection*" OR "Adenovir* Infection?")) OR (TITLE-ABS-KEY ("Human bocavirus*")) OR (TITLE-ABS-KEY ("Hemadsorption Virus 2" OR "Human parainfluenza virus 1" OR "Parainfluenza Virus Type 1" OR "Para-Influenza Virus Type 1" OR "Croup Associated Virus" OR "Acute Laryngotracheobronchitis Virus*" OR "Para-Influenza Virus Type 2" OR "Acute Laryngotracheo-Bronchitis Virus*" OR "Human parainfluenza virus 2" OR "Hemadsorption Virus 1" OR "Human parainfluenza virus 3" OR "Parainfluenza Virus Type 3" OR "Para-Influenza Virus Type 3" OR "Human parainfluenza virus 4" OR "parainfluenza virus type 4" OR "para-influenza

© 2025 Hersi L et al. *JAMA Network Open*.

virus type 4"))) AND ((TITLE-ABS-KEY (("Severe Acute Respiratory Infect*" OR sari OR sentinel) AND ("case definition*" OR case OR cases OR incidence OR epidemiology OR "test* characteristic?" OR "clinical* characteristic?" OR evaluat*))) AND ((TITLE-ABS-KEY ("Multiplex Polymerase Chain Reaction*" OR "Multiplex PCR" OR mpcr OR "Triplex Polymerase Chain Reaction*" OR "Multiplex Ligation-Dependent Probe Amplification")) OR (TITLE-ABS-KEY ((sentinel* OR biosurveillance OR "bio-surveillance") AND ("health event?" OR site OR sites OR illness OR system? OR surveill* OR diagnos* OR "positive predictive value" OR sensitivity OR specificity)))))) AND PUBYEAR > 2024 AND PUBYEAR < 2025

WHO Global Index Medicus March 31, 2025
N=6
(("Severe Acute Respiratory Infect*" or SARI or sentinel) and ("case definition*" or case or cases or incidence or epidemiology or "test* characteristic?" or "clinical* characteristic?" or evaluat*))
AND
("infant disease*" or "childhood disease*" or adolescen* or babies or baby or boy? or boyhood or girlhood or child* or girl? or infan* or juvenil* or kid? or minors or minors* or neonat* or neo-nat* or newborn* or new-born* or paediatric* or peadiatric* or pediatric* or perinat* or preschool* or puber* or pubescen* or school* or teen* or toddler? or underage? or under-age? or youth*)

© 2025 Hersi L et al. *JAMA Network Open*.

eFigure 1. PRISMA Flowchart of Study Screening

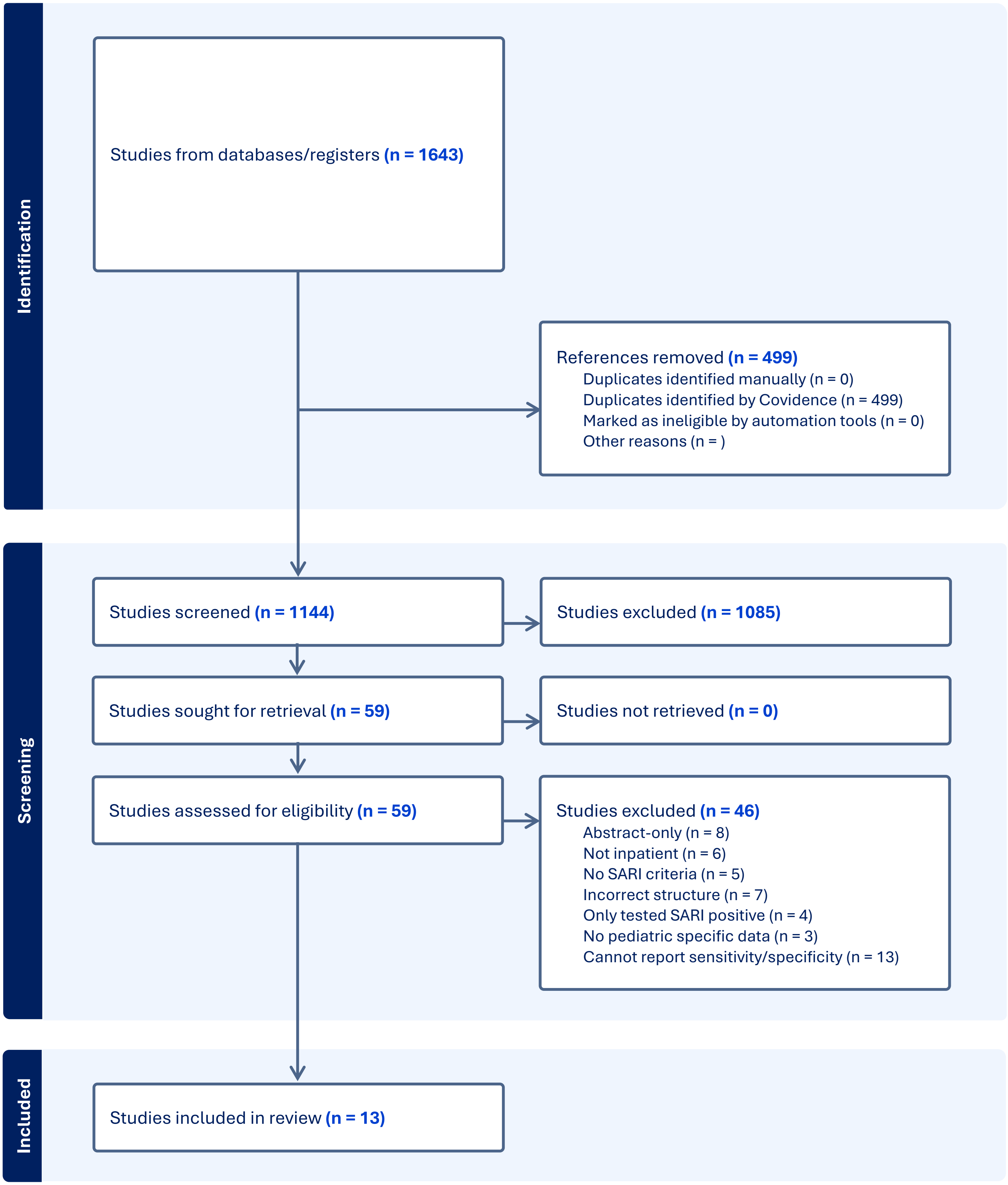

© 2025 Hersi L et al. *JAMA Network Open*.

eTable 1. Risk of Bias Assessment by QUADAS-2 of Included Studies

Reference	Domain 1: Patient Selection		Domain 2: Index Test(s)		Domain 3: Reference Standard		Domain 4: Flow and Timing	
	A: Risk of Bias	B: Applicability	A: Risk of Bias	B: Applicability	A: Risk of Bias	B: Applicability	A: Risk of Bias	
GUPTA ET AL[27], 2013								
MURRAY ET AL[29], 2013								
SAHA ET AL[25], 2015								
JONES ET AL[26], 2016								
MAKOKHA ET AL[30], 2016								
NYAWANDA ET AL[16], 2016								
AMINI ET AL[8], 2017								
NGOBENI ET AL[31], 2019								
RHA ET AL[32], 2019								
KLINK ET AL[14], 2020								
ROWLINSON ET AL[10], 2021								
DAVIS ET AL[9], 2022								
GILL ET AL[28], 2025								

Judgement: Green = Low concern, Yellow = Unclear, Red = High concern

© 2025 Hersi L et al. *JAMA Network Open*.

eTable 2. Meta-Regression Associations Between Prevalence and Test Accuracy (Sensitivity and Specificity) Statistics

Case definition	Virus	Test accuracy statistic	Beta (95% CI)	SE	*P*-value
WHO SARI 2014[1]	Influenza	Sensitivity	1.13 (0.62 to 1.64)	0.26	**<0.001**
		Specificity	-0.83 (-1.33 to -0.32)	0.26	**<0.001**
WHO SARI 2014[2]	RSV	Sensitivity	0.87 (0.30 to 1.45)	0.29	**0.003**
		Specificity	-0.46 (-0.94 to 0.02)	0.24	0.06
IMCI Severe Pneumonia[3]	Influenza	Sensitivity	0.32 (0.03 to 0.60)	0.15	**0.03**
		Specificity	-0.77 (-1.05 to -0.49)	0.14	**<0.001**

[1]Estimates from n=7 studies with influenza prevalence of 3.8% (Klink 2020), 5.3% (Ngobeni 2019), 6.0% (Mokokha 2016), 6.4% (Gill 2025), 9.0% (Rowlinson 2021), 11.9% (Davis 2022), and 20.2% (Amini 2017).

[2]Estimates from n=5 studies with RSV prevalence of 12.3% (Nyawanda 2016), 27.3% (Rha 2019), 41.5% (Davis 2022), 41.8% (Gill 2025), and 44.1% (Klink 2020).

[3]Estimates from n=4 studies with influenza prevalence of 6.0% (Mokokha 2016), 6.8% (Murray 2013), 7.0% (Ngobeni 2019), and 9.0% (Rowlinson 2021).

© 2025 Hersi L et al. *JAMA Network Open*.

eFigure 2. Publication Bias Plots of Diagnostic Accuracy Pooled Analysis

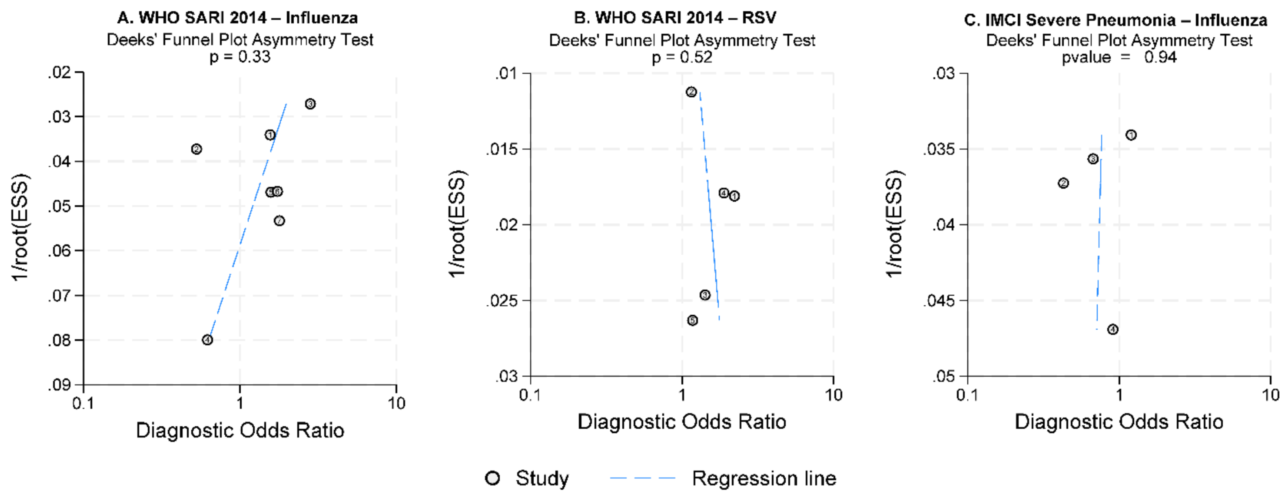

© 2025 Hersi L et al. *JAMA Network Open*.

eTable 3. Diagnostic Accuracy of WHO 2014 SARI Definition Against Other Viruses

	Gill et al [28].			***Klink et al [14].***		
	**Virus positive (%)**	**Sensitivity (%)**	**Specificity (%)**	**Virus positive (%)**	**Sensitivity (%)**	**Specificity (%)**
Enterovirus/Rhinovirus	42.2	34.40	24.94	39.10	36.94	58.28
Adenovirus	31.4	11.99	21.04	14.98	43.67	60.82
Parainfluenza	46.3	15.18	5.17	8.63	58.61	61.92
Human metapneumovirus	44.6	13.22	6.03	5.53	44.00	60.39
SARS-CoV-2	40.9	7.46	4.86			
Seasonal coronavirus	43.1	6.93	3.50			
Bocavirus	43.3	4.76	1.50			

© 2025 Hersi L et al. *JAMA Network Open*.

eTable 4. Age Sub-Grouped Diagnostic Accuracy of SARI Case Definitions in Included Studies

	Virus(es) of interest	Age sub-group	Virus positive (%)	Case definition(s) used	SARI Positive (%)	Sensitivity (%)	Specificity (%)
**DAVIS ET AL[9], 2022**	Influenza	< 3 months	8.2	WHO 2014 SARI	59.7	74.1	41.6
		< 1 year	10.1		75.7	87.4	25.6
		≥ 1 year	14.8		85.6	94.7	16.0
	RSV	< 3 months	48.8	WHO 2014 SARI	59.5	62.4	43.3
		< 1 year	45.0		75.7	81.5	21.1
		≥ 1 year	36.1		85.4	96.4	20.8
**AMINI ET AL[8], 2017**	Influenza	< 1 year	19.2	WHO 2014 SARI	66.9	52.0	29.5
		≥ 1 year	21.2		80.3	79.2	19.1
**RHA ET AL[32], 2019**	RSV	< 3 months	34.0	WHO 2014 SARI	46.6	50.9	55.6
		< 1 year	31.6		58.0	60.2	43.0
		≥ 1 year	19.7		67.0	73.7	34.6
**NYAWANDA ET AL[16], 2016**	RSV	< 1 year	13.6	WHO 2014 SARI	77.7	79.4	22.6
				IMCI Severe Pneumonia	48.0	49.2	52.2
		≥ 1 year	11.3	WHO 2014 SARI	78.1	86.2	22.9
				IMCI Severe Pneumonia	63.3	51.3	45.5
**KLINK ET AL[14], 2020**	Influenza	< 3 months	3.1	WHO 2014 SARI	19.3	15.9	80.6
		< 1 year	3.2		36.0	44.2	64.3
		≥ 1 year	6.4		61.1	75.8	39.9
	RSV	< 3 months	43.1	WHO 2014 SARI	19.3	25.0	85.1
		< 1 year	45.9		36.0	44.3	71.2
		≥ 1 year	34.2		61.1	77.8	47.5
**GILL ET AL[28], 2025**	Influenza	< 3 months	3.1	WHO 2014 SARI	28.3	44.4	72.2
		< 1 year	5.0		47.7	65.5	53.3
		≥ 1 year	7.2		66.3	73.8	34.3
	RSV	< 3 months	77.0	WHO 2014 SARI	23.6	23.9	77.7
		< 1 year	57.3		48.4	50.2	53.9
		≥ 1 year	31.5		65.7	74.3	39.2

© 2025 Hersi L et al. *JAMA Network Open*.

eFigure 3. Forest Plots of Age Sub-Grouped Analysis

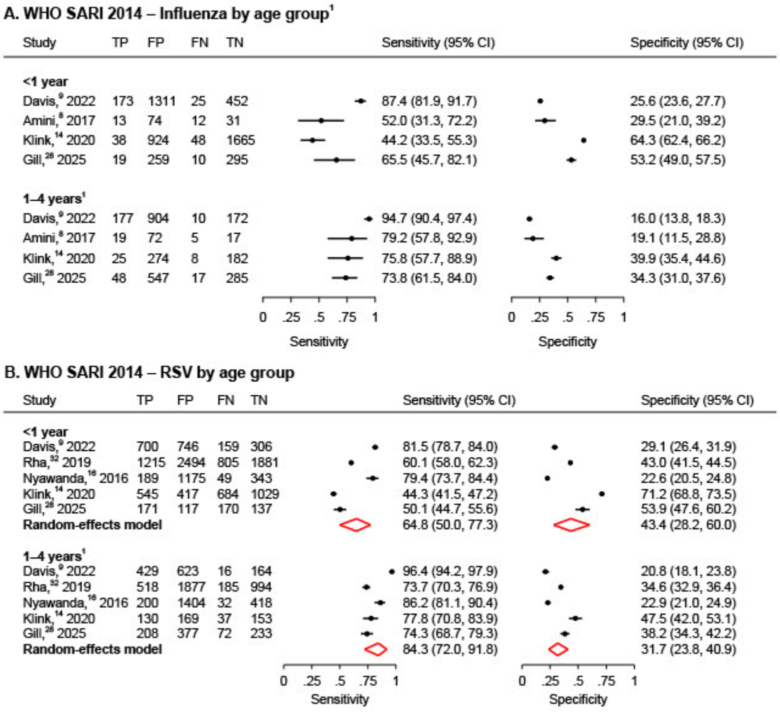

[1]Pooled sensitivity and specificity of the WHO SARI 2014 definition for influenza are not presented, given excessive heterogeneity in hierarchical summary receiver operating curves among subgroups.

© 2025 Hersi L et al. *JAMA Network Open*.
