# Supplementary material for: Diagnostic Accuracy of Severe Acute Respiratory Infection Definitions in Hospitalized Children: A Systematic Review and Meta-Analysis
Source: JAMA Netw Open. 2025 Dec 18;8(12):e2550298. doi: 10.1001/jamanetworkopen.2025.50298 (PMC12715654; doi:10.1001/jamanetworkopen.2025.50298)
Supplement: Supplement 2. — Data Sharing Statement [file jamanetwopen-e2550298-s002.pdf]

Data Sharing Statement

Hersi. Diagnostic Accuracy of Severe Acute Respiratory Infection Definitions in Hospitalized Children. *JAMA Netw Open*. Published December 18, 2025.
doi:10.1001/jamanetworkopen.2025.50298

Data
Data available: Yes
Data types: Deidentified participant data, Data (not involving human participants)
How to access data: Published data from studies included in systematic review
When available: With publication

Supporting Documents
Document types: None

Additional Information
Who can access the data: Anyone requesting the data
Types of analyses: For any purpose
Mechanisms of data availability: With investigator support
